# Long-Term Survival After Mitral Valve Replacement by Preoperative Risk: Implications for Patient Selection

**DOI:** 10.1016/j.athoracsur.2025.07.031

**Published:** 2025-08-14

**Authors:** Matthew Kazaleh, Catherine Wagner, J’undra Pegues, Jessica K. Millar, Carol Ling, Jeremy Wolverton, Rhea Gupta, Robert Hawkins, Steven F. Bolling, Gorav Ailawadi

**Affiliations:** 1 Department of Cardiac Surgery, University of Michigan, Ann Arbor, Michigan; 2 Department of Surgery, University of Michigan, Ann Arbor, Michigan

## Abstract

**BACKGROUND:**

Mitral valve replacement (MVR) is recognized for its significant operative risks. This risk is commonly quantified with The Society of Thoracic Surgeons (STS) Risk Calculator. The present study aimed to investigate the long-term (5-year) predictive capabilities of the STS predicted risk of mortality (PROM) in patients undergoing isolated MVR.

**METHODS:**

Patients undergoing isolated MVR at a high-volume mitral reference center from 2010–2023 were stratified by STS PROM (low: <3%, intermediate: 3%–6%, and high/extreme: >6%). Primary endpoints were short-term (30-day) and long-term (5-year) mortality. Kaplan-Meier survival analysis was used to compare short- and long-term survival among risk stratified groups. Cox regression modeling was used to identify predictors of long-term survival.

**RESULTS:**

Of 1444 of MVR patients, 855 patients underwent isolated MVR. The mean STS PROM for low, intermediate, and high/extreme-risk groups was 1.7%, 4.3%, and 12.7%, respectively. Thirty-day mortality was lower than predicted in the low (0.3%) and high/extreme risk (10.9%) groups. Five-year survival was 90.8%, 75.5%, and 56.4% respectively. Kaplan-Meier survival analysis at 5 years identified significant long-term survival probability differences among the PROM groups, even after accounting for perioperative risk (*P* < .001). Preoperative dialysis (hazard ratio [HR], 1.70; 95% CI 1.05–2.73; *P* = .03), hypertension (HR, 1.71; 95% CI, 1.13–2.59; *P* = .011), and urgent operative status (HR, 1.57; 95% CI, 1.09–2.26; *P* = .02) were among the characteristics associated with worse long-term survival.

**CONCLUSIONS:**

Although predictive models may overestimate surgical risk at high-volume mitral centers, the STS PROM correlates with 5-year mortality in isolated MVR. These findings may provide more specific risk stratification criteria, identifying patients best suited for operative intervention.

Mitral valve replacement (MVR) is recognized for its significant operative risks, leading many high-risk patients to be excluded from surgical management.^1,2^ As transcatheter therapies advance, the importance of surgical risk stratification and patient selection has grown considerably. Surgeons may utilize various tools to evaluate potential surgical risk, most commonly The Society of Thoracic Surgeons (STS) Risk Calculator.^3^ Composed of a robust and evolving calculation of patient specific variables, the STS Risk Calculator allows for predictions of major morbidity, operative mortality, and short-term (30-day) outcomes.

While the STS Risk Calculator is well-established for predicting short-term mortality (predicted risk of mortality [PROM]), its association with long-term mortality remains less understood.^4,5^ Emerging studies have suggested that PROM may be a reliable independent predictor of long-term survival in cardiac surgery, perhaps even up to 14 years after surgery.^6,7^ Although foundational, these studies include a diverse range of cardiac procedures with varying operative risk and limited numbers of mitral operations. Further work is needed to examine patient- and hospital-specific factors that may influence survival rates in isolated mitral surgery.

Accurate short- and long-term risk stratification of patients needing MVR could aid in the identification of those more suitable for less-invasive transcatheter interventions. The present study aimed to investigate the long-term (5-year) predictive capabilities of the STS PROM in patients undergoing isolated MVR at a high-volume mitral reference center. Secondarily, we sought to identify independent predictors of long-term survival in these patients. Finally, we aimed to assess the short-term (30-day) predictive accuracy of PROM for isolated MVR.

## PATIENTS AND METHODS

### STUDY POPULATION.

This study was approved by the University of Michigan institutional review board (HUM00148119). Data from all patients who underwent MVR from 2010 to 2023 (N = 1444), at a single high-volume mitral reference center, were extracted from the STS institutional database. Isolated MVR was defined as MVR procedures with or without tricuspid valve repair, left atrial appendage exclusion and surgical ablation for atrial fibrillation, and/or atrial septal defect repair. Patients who underwent concomitant aortic valve and coronary artery bypass procedures were excluded from analysis. Considering the evolving definition of isolated MVR during this analysis period (including the addition of concomitant tricuspid valve repair), all patients who met the most current criteria for isolated MVR had a PROM calculation performed using all available variables with the STS Risk Calculator (version 2.0.5). A total of 855 patients were identified and subsequently stratified into tertiles by PROM. Low risk was defined as a PROM <3% (n = 319), intermediate 3%–6% (n = 276), and high/extreme >6% (n = 260).

### OUTCOMES.

The primary endpoints of this study were short-term (30-day) and long-term (5-year) mortality, acquired through use of the institutional electronic medical record, the institutional STS database, and the Michigan Death Index.^8^ Secondary outcomes included identification of independent pre- and postoperative patient and hospital specific predictors of long-term survival.

### STATISTICAL ANALYSIS.

Patient characteristics and postoperative complications were presented as counts (%) for categorical variables and median (interquartile range [IQR], 25th-75th percentile) for continuous variables. Differences among groups were evaluated using the χ^2^ test for categorical variables and the Kruskal-Wallis test for continuous variables. Kaplan-Meier survival analysis was used to compare short-term and long-term survival among groups stratified by risk, including a separate landmark analysis at 30 days. Finally, Cox proportional hazards regression models identified independent predictors of long-term survival. All statistical analysis were preformed using R (version 4.3.2; www.r-project.org) with level of significance set at α < 0.05.

## RESULTS

### PATIENT CHARACTERISTICS.

The median age of the entire cohort was 64 (IQR, 54–73) years, including 63.2% female and 81.6% White. The majority (75.5%) of operations was elective, while 23.0% were urgent status. History of previous myocardial infarction was seen in 30.5%, and previous diagnosis of heart failure (New York Heart Association class I-IV) was present in 57.3%. Overall, a cross-clamp was used in 71.5% of cases, with a median cross-clamp time of 97 (IQR, 74–122) minutes and a median cardiopulmonary bypass time of 112 (IQR, 84–149) minutes. A combined 67.6% of patients had moderate or worse mitral regurgitation prior to surgical intervention. Mean (SD) PROM of the entire population was 5.9% (6.54%). An extensive summary of patient characteristics can be seen in Table 1.

Several key differences among groups existed. First, mean (SD) PROM for low, intermediate, and high/extreme risk groups was 1.7% (0.7%), 4.3% (0.8%), and 12.7% (8.3%), respectively. The low risk group was younger than the intermediate and high/extreme risk groups (low: 56 [IQR, 47–64] years, intermediate: 69 [IQR, 59–75] years, high/extreme: 70 [IQR, 60–78] years; *P* < .001). High risk patients were more likely to have urgent operation compared with the low and intermediate risk groups (low: 7.6%, intermediate: 23.9%, high/extreme: 40.8%; *P* < .001). As expected, there were several differences in the prevalence of comorbid disease. With each risk group, there was a higher burden of preoperative comorbidities, including hypertension (58.9% vs 81.9% vs 88.8%; *P* < .001), diabetes (19.7% vs 30.4 % vs 40.8%; *P* < .001), history of previous myocardial infarction (23.2% vs 30.1% vs 40.0%; *P* < .001), and heart failure (50.8% vs 59.8% vs 62.7%; *P* < .001).

No differences in sex, operative approach (sternotomy vs thoracotomy), number of previous cardiac operations, median cross-clamp time, or mitral regurgitation severity were identified. Interestingly, differences in the proportion of rheumatic mitral disease (33.2% vs 23.6% vs 18.8%; *P* < .001), as well as endocarditis (5.6% vs 9.8% vs 14.6%; *P* = .001) were seen. Notably, differences in the proportion of concomitant tricuspid valve repair were also observed across groups (27.9% vs 40.9% vs 41.9%; *P* < .001). A comprehensive list of isolated MVR procedure specifics can be found in Table 2.

### POSTOPERATIVE OUTCOMES.

Following isolated MVR, the low and intermediate risk groups had significantly shorter total intensive care unit lengths of stay (hours) (low: 52 [IQR, 29–95], intermediate: 95 [IQR, 48–159], high/extreme: 121 [IQR, 72–203]; *P* < .001), as well as total hospital lengths of stay (days) (low: 8 [IQR, 5–13], intermediate: 12 [IQR, 7–21], high/extreme: 17 [IQR, 10–28]; *P* < .001), compared to the high/extreme risk group. Furthermore, the high/extreme risk group experienced significantly more postoperative complications, such as prolonged ventilation (low: 6.9% vs intermediate: 18.1% vs high/extreme: 36.5%; *P* < .001), postoperative pneumonia (low: 2.2% vs intermediate: 8.0% vs high/extreme: 12.3%; *P* < .001), and renal failure (low: 1.9% vs intermediate: 4.7% vs high/extreme: 13.5%; *P* < .001). Overall, the majority of patients (74.2%) was discharged home, although a higher proportion of intermediate and high/extreme risk patients were sent to extended care rehabilitation facilities. A summary of postoperative outcomes can be seen in Table 3.

### SHORT-TERM SURVIVAL.

**FIGURE 1:**
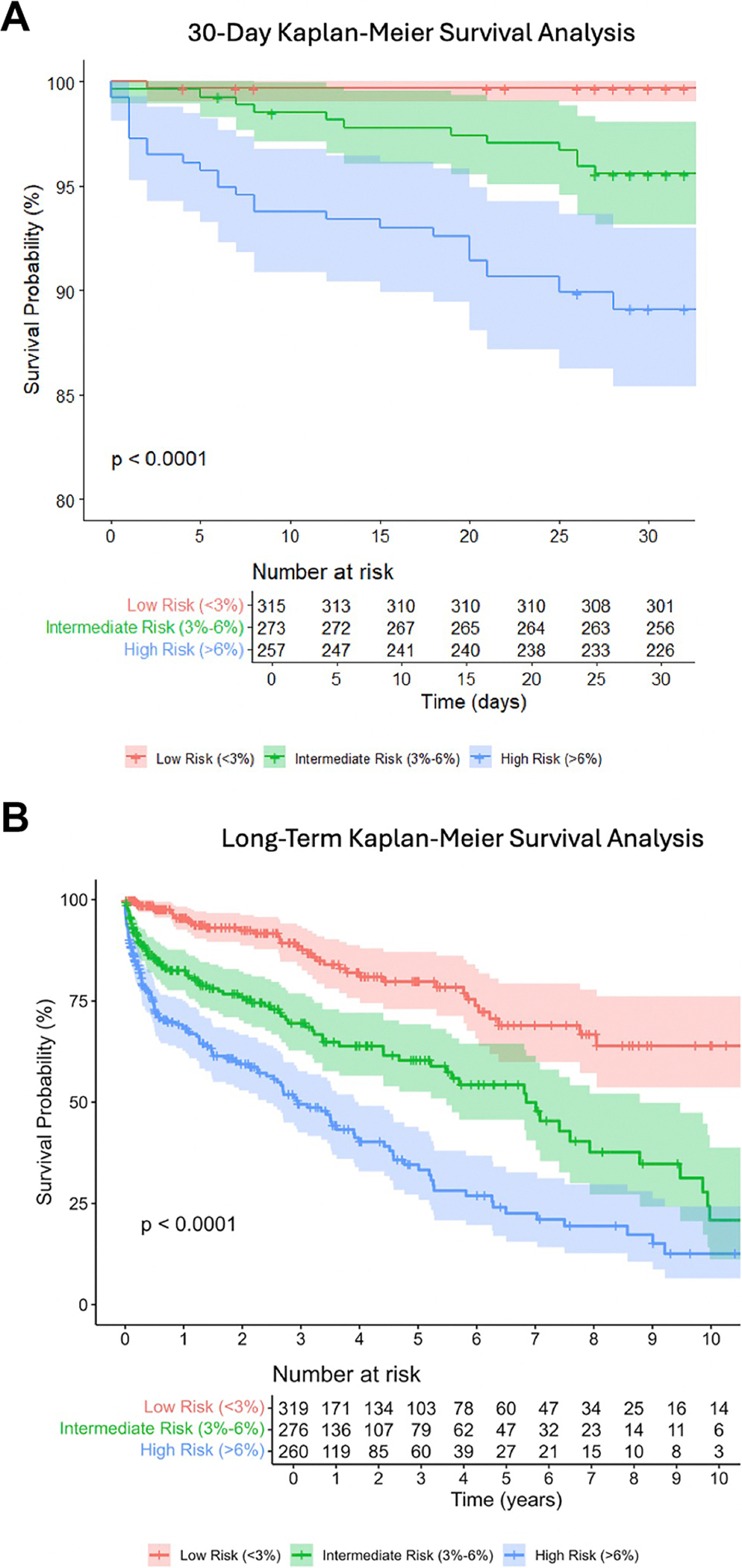
(A) Short-term and (B) long-term Kaplan-Meier survival analysis by predicted risk of mortality groups.

The observed to expected 30-day mortality was lower in the low and high/extreme risk groups, respectively (0.3% vs 1.7%; 10.9% vs 12.7%). In the intermediate risk group, the observed 30-day mortality nearly matched expected (4.3% vs 4.4%). A summary of short- and long-term mortality rates are presented in Table 4. Kaplan-Meier survival analysis at 30 days revealed significant differences in survival probabilities among our PROM groups, particularly in the high/extreme risk group (*P* < .001) (Figure 1A). Notably, after 15 days, predicted survival differences of the low and intermediate risk groups became more pronounced.

### LATE SURVIVAL.

Mean follow-up time was 2.26 years (95% CI, 1.96–2.55; *P* < .001), and did not differ among groups. Survival at 5 years was 90.8% in the low risk group, 75.5% in the intermediate risk group, and 56.4% in the high/extreme risk group. Additional information regarding proportion of known follow-up can be found in Supplemental Table 1. Kaplan-Meier survival analysis at 5 years identified significant long-term survival probability differences among the PROM groups (*P* < .001) (Figure 1B). Higher PROM was associated with worse long-term survival, even after adjusting for perioperative risk using a 30-day land-mark survival analysis (*P* < .001) (Supplemental Figure 1).

### MULTIVARIABLE ANALYSIS.

**FIGURE 2:**
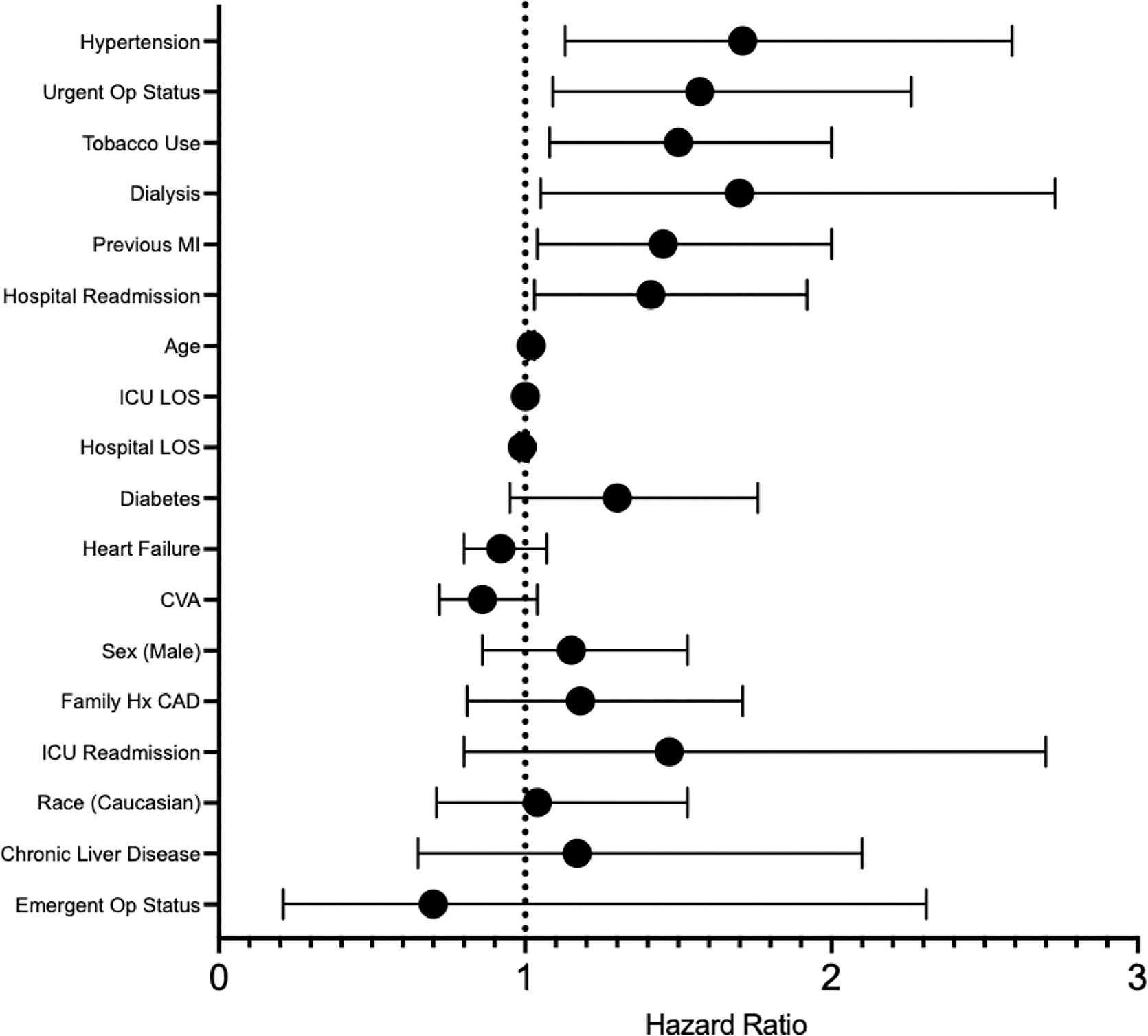
Hazard ratios forest plot for patient- and hospital-specific characteristics as independent predictors of survival in isolated mitral valve replacement. Heart failure defined as NewYork Heart Association Class I-IV. (CAD, coronary artery disease; CVA, cerebrovascular accident; Hx, history; ICU, intensive care unit; LOS, length of stay; MI, myocardial infarction; Op, operative.)

Cox proportional hazards regression models identified several patient characteristics associated with worse long-term survival. Age (hazard ratio [HR], 1.02; 95% CI, 1.01–1.03; *P* = .001), preoperative dialysis (HR, 1.70; 95% CI, 1.05–2.73; *P* = .030), hypertension (HR, 1.71; 95% CI, 1.13–2.59; *P* = .011), urgent operative status (HR, 1.57; 95% CI, 1.09–2.26; *P* = .016), and tobacco use (HR, 1.47; 95% CI, 1.08–2.00; *P* = .015) were among the preoperative characteristics associated with worse long-term survival. Interestingly, heart failure, chronic liver disease, history of stroke, and emergent operative status were not associated with worse survival in our models. Postoperatively, hospital readmission (HR, 1.41; 95% CI, 1.03–1.92; *P* = .032) was associated with worse survival. A forest plot of patient and hospital specific characteristics as independent predictors of survival can be found in Figure 2 with a comprehensive list in Supplemental Table 2.

## COMMENT

The present study evaluated outcomes of isolated MVR as well as the association of STS PROM with operative mortality and late survival. First, we identified significant 5-year survival probability differences of three risk stratified PROM categories, even after accounting for perioperative risk. Higher PROM was associated with worse long-term survival. Second, we identified several patient- and hospital-specific risk factors associated with worse long-term survival, including hypertension, urgent operative status, preoperative dialysis, and hospital readmission. Third, we found that the short-term (30-day) predictive capability of PROM may overestimate observed mortality at a high-volume mitral center, particularly for low and high risk patients.

The emergence of transcatheter MVR (TMVR) has highlighted the growing importance of accurately stratifying surgical risk. Numerous clinical trials have explored the potential benefits of TMVR, with the most notable being improved early survival in higher-risk patients.^9^ However, despite this short-term mortality benefit, concerns persist regarding the durability and long-term outcomes of TMVR.^10^ There remains a patient population that could still benefit from surgical MVR, but an accurate method for identifying this group has yet to be established. Based on the notable differences in long-term survival probabilities observed with our defined PROM risk categories (low <3%, intermediate 3%–6%, high/extreme >6%), we propose that these groups could effectively classify long-term risk, pending further detailed validation. Clearly defined risk-stratified groups with significant survival differences could assist in determining candidacy for isolated MVR, facilitating more informed patient decision-making that considers both short- and long-term outcomes.

Previous studies have sought to elucidate the relationship between PROM and long-term survival in cardiac surgery. One of the most robust investigations explored this association among 24,222 qualifying PROM procedures at a single academic center from 1996 to 2009.^6^ The group found that among those who survived the first 30 days, each percentage-point increase in PROM score corresponded to a 9.6% increase (95% CI, 9.3–10.0; *P* < .001) in the instantaneous hazard of death. The authors suggest that this association continued long term, with the predictive ability of PROM persisting for up to 14 years postoperatively. That study, however, included all types of operations and did not separate MVR from mitral valve repair. Given the well-established differences in all-cause long-term morbidity and mortality between MVR and mitral valve repair, our study extends this research by confirming these findings specifically in patients undergoing isolated MVR.

Integral in this patient selection is the identification of patient- and hospital-specific predictive factors contributing to worse survival after mitral valve operations.^7,11^ In their investigation involving 1256 patients undergoing PROM qualifying procedures, Ben-David and associates^7^ identified several risk factors associated with diminished long-term survival. These included age over 65 years (HR, 1.6; 95% CI, 1.1–2.3; *P* = .02), preoperative dialysis (HR, 2.5; 95% CI, 1.2–4.9; *P* = .01), diabetes (HR, 1.5; 95% CI, 1.1–2.0; *P* = .02), and increased PROM. Notably, their study encompassed all PROM qualifying procedures at a single hospital, with MVR and mitral valve repair cases constituting only 10%–14% of all procedures. Our study further refines these findings by focusing on the highest-risk STS procedure—isolated MVR—at a high-volume mitral referral center. Our results reaffirm the association of factors like advanced age and preoperative dialysis with worse long-term survival, while also identifying additional predictive variables such as hypertension, tobacco use, previous myocardial infarction, and urgent operative status. Such predictors could aid in the identification of patients at risk for increased morbidity and mortality.

Interestingly, a strong association between higher hospital volume of mitral valve surgeries and lower adjusted operative morbidity and mortality has been established.^12–14^ For example, Gammie and colleagues^12^ reported a robust, multicenter retrospective analysis of elective mitral operations conducted between 2000 and 2003. After adjustment for preoperative clinical risk, rates of operative mortality decreased with increasing mitral surgery volume, specifically with hospital volume of greater than 140 mitral surgeries per year (odds ratio, 0.48; 95% CI, 0.28–0.82; *P* = .0043). This volume-mortality relationship remained significant even when accounting for the proportion of MVR vs mitral valve repair procedures. In our study, we observed less mortality than expected in our low and high risk patients, at 30 days. Given the established correlation between hospital volume and reduced mortality in mitral surgery, it is possible that PROM calculations may overestimate mortality risks for patients undergoing isolated MVR at high-volume centers. At 30 days, notable differences in survival probability existed among our defined PROM categories, although they were most pronounced in high/extreme risk patients. Moreover, the high/extreme risk cohort had dismal long-term predicted survival (43.58% at 5 years), even in a high-volume mitral center, suggestive that surgical alternatives may have been appropriate.

This study should be interpreted in the context of key limitations. First, the authors recognize that the STS Risk Calculator is an evolving algorithm, with potential fluctuations in accuracy and variable composition throughout the study period. Modifiers were not used in this analysis. Values for PROM group stratification were selected based upon establishment of equal cohort size, with previous literature providing clues as to where clinically significant boundaries may exist.^15^ Although well-defined long-term survival differences were established, further detailed stratification could allow for more specific predicted survival. Additionally, this work only includes patients who were offered and received an operation. Those who were turned down as poor surgical candidates were not captured in our model. Further work could incorporate several cardiac surgery risk models for an accurate risk assessment in isolated MVR. Additional studies could also attempt longer survival analysis, especially with a more diverse patient population with longer patient follow-up, as our study consisted of a mean follow-up time of 2.26 years and was powered enough to provide statistical significance to only 5 years. Finally, it is important to recognize that the generalizability of our findings may only extend to other high-volume mitral valve reference centers, as volume and complexity of disease may influence outcomes, risk stratification, and surgical decision-making.

In conclusion, these findings suggest that the STS Risk Calculator short-term PROM correlates with 5-year mortality in isolated mitral valve replacement. Several patient- and hospital-specific risk factors were identified and associated with worse long-term survival. These findings collectively offer more refined risk stratification criteria to help identify patients who are candidates for isolated mitral valve replacement and those who may be better suited pursuing alternative therapies.

## Supplementary Material

1

The Supplemental Material can be viewed in the online version of this article [https://doi.org/10.1016/j.athoracsur.2025.07.031] on http://www.annalsthoracicsurgery.org.

## Figures and Tables

**TABLE 1:** Preoperative Characteristic by Society of Thoracic Surgeons Risk Calculator Predicted Risk of Mortality (PROM)

Variable	Overall	PROM <3%	PROM 3%-6%	PROM >6%	*P* Value

N	855	319	276	260	
Age, y	64 (54–73)	56 (47–64)	69 (59–75)	70 (60–78)	<.001
Cross-clamp time, min	97 (74–122)	94 (68–121)	99 (78–125)	94 (77–122)	.226
Sex					
Male	315 (36.8)	122 (38.2)	107 (38.8)	86 (33.1)	.412
Female	540 (63.2)	197 (61.8)	169 (61.2)	174 (66.9)	.318
Race^a^					
White	690 (81.6)	252 (79.0)	232 (84.1)	206 (79.2)	.265
Non-White	165 (19.3)	67 (21.0)	44 (15.9)	54 (20.8)	
Tobacco use^a^	423 (49.4)	156 (48.9)	140 (50.7)	127 (48.8)	.909
Hypertension	645 (75.4)	188 (58.9)	226 (81.9)	231 (88.8)	<.001
Diabetes	253 (29.6)	63 (19.7)	84 (30.4)	106 (40.8)	<.001
Chronic liver disease	60 (7.0)	16 (5.0)	15 (5.4)	29 (11.2)	.007
Chronic kidney disease	54 (6.3)	6 (1.9)	13 (4.7)	35 (13.5)	<.001
Heart failure^a^	489 (57.3)	161 (50.8)	165 (59.8)	163 (62.7)	<.001
Atrial fibrillation^c^					<.001
None	314 (46.7)	150 (58.4)	89 (42.0)	75 (36.9)	
Persistent	182 (27.1)	44 (17.1)	72 (34.0)	66 (32.5)	
Paroxysmal	176 (26.2)	63 (24.5)	51 (24.1)	62 (30.5)	
Previous MI	261 (30.5)	74 (23.2)	83 (30.1)	104 (40.0)	<.001
MR severity^b^					.102
Trace	70 (8.2)	21 (6.6)	20 (7.2)	29 (11.2)	
Mild	150 (17.5)	43 (13.5)	55 (19.9)	52 (20.0)	
Moderate	159 (18.6)	58 (18.2)	51 (18.5)	50 (19.2)	
Severe	419 (49.0)	173 (54.2)	134 (48.6)	112 (43.1)	
Etiology					
Rheumatic	220 (25.7)	106 (33.2)	65 (23.6)	49 (18.8)	<.001
Endocarditis	83 (9.7)	18 (5.6)	27 (9.8)	38 (14.6)	.001
Degenerative	95 (11.1)	28 (8.8)	37 (13.4)	30 (11.5)	.194
Functional	28 (3.3)	6 (1.9)	10 (3.6)	12 (4.6)	.171
MAC	36 (4.2)	7 (2.2)	14 (5.1)	15 (5.8)	.071
Mixed	82 (9.6)	21 (6.6)	31 (11.2)	30 (11.5)	.07
Other	353 (41.3)	144 (45.1)	109 (39.5)	100 (38.5)	.204
Unknown	67 (7.8)	22 (6.9)	20 (7.2)	25 (9.6)	.435
Operative status^a^					<.001
Elective	644 (75.5)	292 (92.1)	210 (76.1)	142 (54.6)	
Urgent	196 (23.0)	24 (7.6)	66 (23.9)	106 (40.8)	
Emergent	12 (1.4)	1 (0.3)	0 (0.0)	11 (4.2)	
Emergent salvage	1 (0.1)	0 (0.0)	0 (0.0)	1 (0.4)	
CSI^a^					.193
1^st^	402 (47.1)	158 (49.8)	131 (47.5)	113 (43.5)	
2^nd^	343 (40.2)	127 (40.1)	114 (41.3)	102 (39.2)	
3^rd^	80 (9.4)	27 (8.5)	22 (8.0)	31 (11.9)	
4^th^	23 (2.7)	5 (1.6)	7 (2.5)	11 (4.2)	
5^th^	5 (0.6)	0 (0.0)	2 (0.7)	3 (1.2)	
Operative approach					.423
Sternotomy	591 (69.1)	223 (69.9)	196 (71.0)	174 (65.2)	
Thoracotomy	250 (29.2)	93 (29.2)	76 (27.5)	85 (31.8)	
Other	14 (1.7)	3 (0.9)	4 (1.5)	8 (3.0)	

a Degree of missingness ≤ 1%;

b degree missingness ≤ 7.5%;

c degree missingness ≤ 22%. Heart failure defined as New York Heart Association Class ≥III. Values reported as median (interquartile range: 25th-75th percentile) or n (%). *P* values based on χ^2^ test for categorical variables and Kruskal-Wallis test for continuous variables. CSI, cardiac surgery incidence (number of previous cardiac operations including current operation); MAC, mitral annulus calcification; MI, myocardial infarction; MR, mitral regurgitation; PROM, predicted risk of mortality.

**TABLE 2:** Isolated Mitral Valve Replacement and Qualifying Procedure Types by PROM Group

Procedure	Overall	PROM <3%	PROM 3%-6%	PROM >6%	*P* Value

N	855	319	276	260	
MVR (only)	390 (45.6)	164 (51.4)	113 (40.9)	113 (43.5)	.027
TVr	311 (36.4)	89 (27.9)	113 (40.9)	109 (41.9)	<.001
AF	253 (29.6)	95 (29.8)	86 (31.2)	72 (27.7)	.677
ASD	59 (6.9)	21 (6.6)	21 (7.6)	17 (6.5)	.853
Multiple	115 (13.5)	38 (11.9)	40 (14.5)	37 (14.2)	.022
All	14 (1.6)	4 (1.3)	6 (2.2)	4 (1.5)	.022

Values represent absolute counts of each procedure reported as n (%); *P* value based on χ^2^ test for categorical variables. AF, atrial fibrillation surgical procedure defined as left atrial appendage exclusion and ablation; ASD, atrial septal defect repair; MVR, mitral valve replacement with no other concomitant procedures; PROM, predicted risk of mortality; TVr, tricuspid valve repair.

**TABLE 3:** Postoperative Outcomes and Complications by PROM Group After Isolated Mitral Valve Replacements

Variable	Overall	PROM <3%	PROM 3%-6%	PROM >6%	*P* Value

N	855	319	276	260	
Total hospital length of stay^a^, d	11 (7–20)	8 (5–13)	12 (7–21)	17 (10–28)	<.001
Total ICU length of stay^a^, h	79 (45–149)	52 (29–95)	95 (48–159)	121 (72–203)	<.001
Prolonged mechanical ventilation	167 (19.5)	22 (6.9)	50 (18.1)	95 (36.5)	<.001
Pneumonia	61 (7.1)	7 (2.2)	22 (8.0)	32 (12.3)	<.001
Renal failure	54 (6.3)	6 (1.9)	13 (4.7)	35 (13.5)	<.001
Hospital readmission^b^	165 (20.4)	50 (15.8)	53 (20.2)	62 (27.0)	.028
ICU readmission^a^	45 (5.3)	13 (4.1)	11 (4.0)	21 (8.1)	.05
Operative mortality	61 (7.1)	2 (0.6)	21 (7.6)	38 (14.6)	<.001
Discharge location^b^					<.001
Extended care facility	195 (24.0)	23 (7.3)	68 (25.8)	104 (45.0)	
Home	602 (74.2)	293 (92.7)	191 (72.3)	118 (51.1)	
Other	14 (1.7)	0 (0)	5 (1.9)	9 (3.9)	

a Degree of missingness ≤1%;

b degree of missingness ≤5%. Prolonged mechanical ventilation defined by STS as postoperative pulmonary ventilation >24 hours; operative mortality defined as all deaths, regardless of cause, occurring during the hospitalization in which operation was performed, even after 30 days (including patients transferred to other acute care facilities), and all deaths occurring prior to 30 days. Values reported as median (interquartile range: 25th-75th percentile) or n (%). *P* values based on χ^2^ test for categorical variables and Kruskal-Wallis test for continuous variables. ICU, intensive care unit; PROM, predicted risk of mortality.

**TABLE 4:** Isolated Mitral Valve Replacement Observed Mortality by PROM Group

	Observed Mortality					
Group	Mean PROM	30 Day	1 Year	3 Year	5 Year	10 Year

PROM <3%	1.70 (0.71)	0.32	3.17	6.35	9.21	12.06
PROM 3%-6%	4.30 (0.83)	4.40	14.65	21.25	24.54	30.04
PROM >6%	12.68 (8.27)	10.89	27.63	37.74	43.58	49.03

Values are presented as percent. Mean PROM reported as mean (SD). PROM, predicted risk of mortality.
