# Supplementary material for: Long-Term Survival After Mitral Valve Replacement by Preoperative Risk: Implications for Patient Selection
Source: Ann Thorac Surg. Author manuscript; Available in PMC 2026 Aug 2. (PMC13429166; doi:10.1016/j.athoracsur.2025.07.031)
Supplement: 1 [file NIHMS2181299-supplement-1.docx]

**Supplemental:**

**Supplemental Table 1.** Percentage of Known Fate/Follow Up Following Mitral Valve Replacement

| Follow Up 1-Year Minimum | Follow Up 3-Year Minimum | Follow Up 5-Year Minimum | Follow Up 10-Year Minimum |
| --- | --- | --- | --- |
| 64.4% | 49.4% | 44.7% | 32.1% |

*Percent follow up values include both known mortality and survival

**Supplemental Figure 1.** Landmark (30-day) Kaplan-Meier Long-Term Survival Analysis by PROM Groups


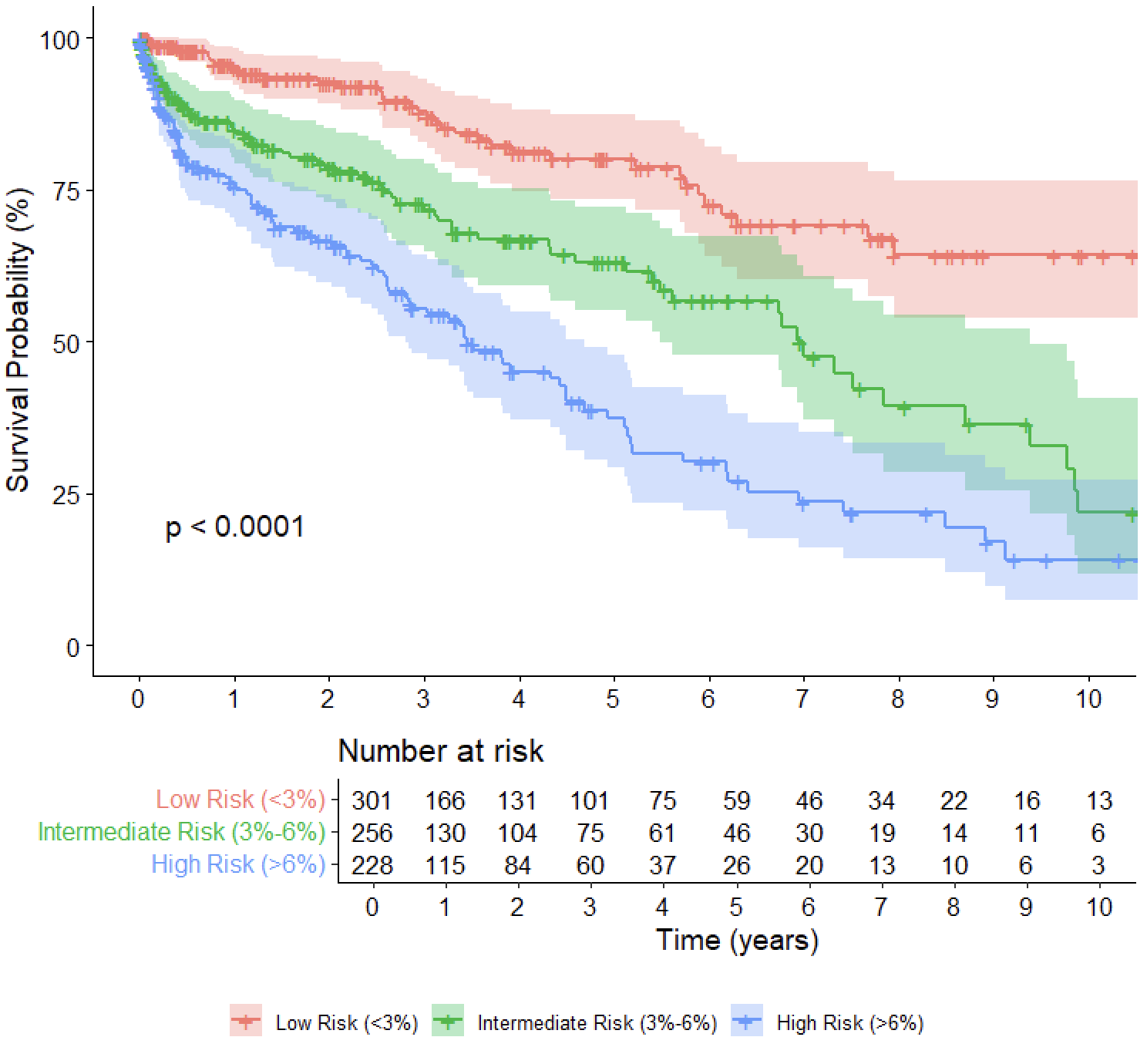


**Supplemental Table 2.** Patient and Hospital Specific Characteristics as Independent Predictors of Survival in Isolated Mitral Valve Replacement

|  | Hazard Ratio |  |  |  |  |
| --- | --- | --- | --- | --- | --- |
| Characteristic | Exp (coef) | Exp (-coef) | Lower .95 | Upper .95 | p-value |
| Age | 1.02 | 0.98 | 1.01 | 1.03 | 0.001* |
| Sex (Male) | 1.15 | 0.87 | 0.86 | 1.53 | 0.354 |
| Race (Caucasian) | 1.04 | 0.96 | 0.71 | 1.54 | 0.825 |
| Emergent Operative Status | 0.70 | 1.42 | 0.21 | 2.31 | 0.562 |
| Urgent Operative Status | 1.57 | 0.64 | 1.09 | 2.26 | 0.016* |
| Heart Failure | 0.92 | 1.08 | 0.80 | 1.07 | 0.287 |
| Chronic Liver Disease | 1.17 | 0.85 | 0.65 | 2.10 | 0.594 |
| Diabetes | 1.30 | 0.77 | 0.95 | 1.76 | 0.099 |
| Dialysis | 1.70 | 0.59 | 1.05 | 2.73 | 0.030* |
| Hypertension | 1.71 | 0.58 | 1.13 | 2.59 | 0.011* |
| Cerebrovascular Accident | 0.86 | 1.16 | 0.72 | 1.04 | 0.118 |
| Previous MI | 1.45 | 0.69 | 1.05 | 1.99 | 0.024* |
| Family History CAD | 1.18 | 0.85 | 0.81 | 1.71 | 0.387 |
| Tobacco Use | 1.47 | 0.68 | 1.08 | 2.00 | 0.015* |
| Total Hospital LOS | 0.99 | 1.01 | 0.98 | 1.01 | 0.436 |
| Total ICU LOS | 1.00 | 1.00 | 1.00 | 1.00 | 0.002* |
| ICU Readmission | 1.47 | 0.68 | 0.80 | 2.70 | 0.214 |
| Hospital Readmission | 1.41 | 0.71 | 1.03 | 1.92 | 0.032* |

Heart Failure defined as New York Heart Association Class I-IV, MI (Myocardial Infarction), CAD (coronary artery disease), LOS (length of stay), ICU (intensive care unit), *significance

Concordance = 0.732 (standard error = 0.019 )

Likelihood ratio test = 110.9 on 19 degrees of freedom, p=5e-15

Wald test = 126.8 on 19 degrees of freedom, p<2e-16

Score (logrank) test = 159.8 on 19 degrees of freedom, p<2e-16
